# Supplementary material for: MetaRibo-Seq measures translation in microbiomes
Source: Nat Commun. 2020 Jun 29;11:3268. doi: 10.1038/s41467-020-17081-z (PMC7324362; doi:10.1038/s41467-020-17081-z)
Supplement: Supplementary file 10 — Supplementary Data 7 [file 41467_2020_17081_MOESM10_ESM.zip › File2/Confidence_High_Taxonomy/108674_out.krona.html]

Javascript must be enabled to view this page.

members
magnitude
magnitudeUnassigned
count
unassigned
taxon
rank

108674\_out

26

superkingdom
26
2

26
phylum
976

200643
class
26

order
26
171549

815
26
family

816

SRS075078\_contig\_number\_21722SRS1041137\_contig\_number\_22991
26
genus
2

1929999

SRS018836\_contig\_number\_21080SRS144506\_contig\_number\_contig-100\_433.272811SRS148196\_contig\_number\_33670
species
3

162156

SRS015890\_contig\_number\_5299SRS018984\_contig\_number\_6621SRS023583\_contig\_number\_17606SRS065176\_contig\_number\_9421SRS077127\_contig\_number\_2946SRS140492\_contig\_number\_52
species
6


SRS012273\_contig\_number\_50118SRS013940\_contig\_number\_22486SRS014948\_contig\_number\_17900SRS015960\_contig\_number\_574SRS018623\_contig\_number\_19260SRS020869\_contig\_number\_13188SRS045645\_contig\_number\_2750SRS056259\_contig\_number\_28550SRS056273\_contig\_number\_18721SRS101376\_contig\_number\_396SRS104636\_contig\_number\_24228SRS142599\_contig\_number\_15785
12
species
329854

species
1

SRS147139\_contig\_number\_17666
457390

246787
2
species

SRS024492\_contig\_number\_19159SRS1041118\_contig\_number\_1924
